# Beyond the Whole: Reduced Empathy for Masked Emotional Faces Is Not Driven by Disrupted Configural Face Processing

**DOI:** 10.3390/bs14090850

**Published:** 2024-09-20

**Authors:** Sarah D. McCrackin, Jelena Ristic

**Affiliations:** Department of Psychology, McGill University, 2001 McGill College Avenue, Montreal, QC H3A 1G1, Canada; jelena.ristic@mcgill.ca

**Keywords:** lower face occlusion, face masks, inversion, configural face perception, affective empathy, facial emotion, facial expressions, face masks

## Abstract

Sharing of emotional states is reduced for individuals wearing face coverings, but the mechanism behind this reduction remains unknown. Here, we investigated if face occlusion by masks reduces empathy by disrupting configural processing of emotional faces. Participants rated their empathy for happy and neutral faces which were presented in upright or inverted orientation and wore opaque, clear, or no face masks. Empathy ratings were reduced for masked faces (opaque or clear) as well as for inverted faces. Importantly, face inversion disrupted empathy more for faces wearing opaque masks relative to those wearing clear or no masks, which stands in contrast to the predictions generated by the classic configural processing models. We discuss these data within the context of classic and novel configural face perception models, and highlight that studying inverted occluded faces presents an informative case worthy of further investigation.

## 1. Introduction

Faces communicate diverse social messages, from basic emotional expressions to complex socioemotional variables like theory of mind [1,2,3]. Visual face occlusion accordingly has been found to produce sizable detriments in social and emotional processing that depend on extraction of information from faces. Face occlusion can occur in many forms, including religious garments and personal protective equipment, and arguably has never been as common as when COVID-19 led to widespread face mask adoption in the general worldwide population. While face masks remain one of the best methods for disease prevention [4,5,6], masked social interactions have been found to create sizable social barriers (e.g., [7,8,9]). That is, lower face occlusion has been found to impair important facets of social processing, including recognition of facial identity [10,11,12], age [11], gender [11], and discrimination of emotional expressions [10,11,13,14], as well as judgements of emotional intensity [15,16] and empathy [15,17]. While this work has revealed new ways in which visual information from faces contributes to human social communication (e.g., [18]), there remains an important outstanding question about the underlying visual and perceptual mechanisms by which facial occlusion affects social function. Here, we addressed this question by investigating if reduced empathy for emotional masked faces occurs because of the disruption of configural face perception.

Our work has demonstrated that lower face occlusion by masks results in significantly lower ratings of positive affective empathy, or sharing of positive emotional states, for mask wearers as opposed to non-mask wearers [15,17]. In this study, participants rated their empathy for happy, neutral, and sad faces that were unoccluded, wearing opaque masks, or wearing clear masks, a manipulation which allowed most of the face to remain visible. Participants reported lower positive empathy for faces with both clear and opaque masks relative to protagonists with unoccluded faces. This result was surprising, since the restoration of the visual input of the emotional face in the clear mask condition was unable to restore empathic responses, suggesting that a mechanism other than basic visual perception may be associated with reduced empathy for occluded faces (see also [19]).

One possibility is that both opaque and clear face masks disrupt configural face processing (e.g., [11,20]), and in turn disrupt the extraction of socioemotional value from faces. Faces are commonly understood to be perceived in a process by which featural perception of the face parts including the eyes, nose, and mouth co-occurs with configural perception of the face as a whole, which combines these features into a unified percept [21]. Face inversion is a popular experimental manipulation which is believed to impair configural face processing while leaving featural face processing intact (e.g., [22,23,24,25]). When faces are upright, participants are found to perform better on face-specific tasks such as face recognition (e.g., [26]) or emotion recognition (e.g., [27]), indicating that configural perception is important for those processes. The difference in performance for upright vs. inverted conditions is often referred to as the Face Inversion Effect, with a larger inversion effect magnitude understood to indicate more configural processing required or occurring [23,28]. For example, larger face inversion effects are found for faces than for objects or individual face parts, which is interpreted to indicate that faces benefit from configural processing in the upright configuration, and this benefit is removed when they are inverted [23,28]. In contrast, responses to objects and face parts are believed to be relatively unaffected by inversion because do they not normally benefit from configural processing when upright [23,28].

Preliminary work suggests that configural face processing disruption resulting from facial occlusion can explain some [12,20], but not all [11], social impairments resulting from face occlusions by face masks. Both Freud et al. [20] and Stajduhar et al. [12] reported larger face inversion effects for unmasked compared to masked faces when participants engaged in a face recognition task. This was interpreted as indicating that face masks disrupt configural processing of upright faces, and as such, recognition of masked faces is less impacted by face inversion than recognition of unmasked faces. However, other research suggests that when faces are judged on social dimensions other than person identity, little or opposite effects of face occlusion by face masks on face inversion are found. Fitousi et al. [11] examined the impact of face masks on emotion, age, and gender discrimination for upright and inverted faces. While face inversion similarly impacted masked and unmasked faces for emotion and age discrimination tasks, a larger face inversion effect was found for faces wearing masks in the gender discrimination task, which contrasts the predictions from the classic configural understanding of face perception. These results suggested that disrupted configural face perception alone likely does not explain why emotion, age, and gender discrimination are impaired when faces are occluded by masks.

A similar comparison of the inversion effects for unmasked and masked faces can be used to investigate whether face masks impair empathic responses through disruption of configural face perception, which is the approach we used in the present work. We presented participants with images of upright and inverted happy and neutral faces wearing no masks, clear masks, or opaque masks. As in McCrackin et al. [15] (see also [17]), participants were asked to rate their empathy and shared emotional valence for the masked and unmasked protagonists. Based on past work, for the upright condition, we expected to observe reduced empathy for protagonists wearing masks, both opaque and clear [15,17]. If this impairment was driven by the disruption of configural face processing by face occlusion via masks, we expected to find more reduction in empathy for inverted faces wearing no masks compared to inverted faces wearing both opaque and clear masks, as, following from the previous literature [12,20], configural processing of unmasked faces should be the most affected by inversion.

## 2. Methods

### 2.1. Participants

A target sample size of 120 participants was determined from an initial pilot study (an initial pre-registered pilot study was completed with a sample of 71. Based on the findings from this study, we updated the pre-registration with this finding and our intent to run a high-powered replication (*n* = 120) using a new sample, which is reported in the present paper) using a separate participant group, and pre-registered alongside the study methods at https://osf.io/vswh3 (accessed on 8 September 2024). One hundred and twenty-five participants (no participants were excluded from analyses based on the pre-registered criteria of over 20% data loss after screening for anticipatory (responses made in less than 500 ms) or missed responses (responses with no rating)) completed the study for course credit (104 women, 18 men, 3 other; mean age: 21.01, *SD* = 3.678). All participants reported English fluency, significant Caucasian face exposure, normal-to-corrected to normal vision, no history of head trauma resulting in loss of consciousness, and no current diagnosis of a mental health condition or cognitive impairment. This study was conducted according to the guidelines of the Declaration of Helsinki, and was approved by the McGill University Research Ethics Board. Informed consent was obtained from all participants involved in the study.

### 2.2. Stimuli

**Figure 1:**
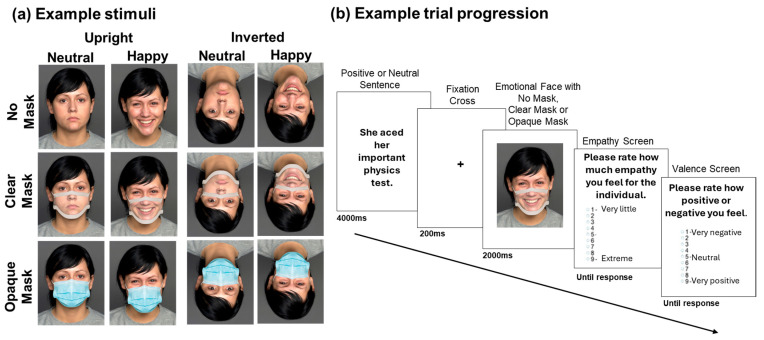
Example of (**a**) neutral and happy upright and inverted face stimuli for each mask condition and (**b**) trial progression depicting a happy clear mask trial.

Happy and neutral face images were retrieved from the FACES [29] and KDEF [30] databases (KDEF Identities: KM08, KM11, KM31, KM35. FACES Identities: F05, F10, F13, F19, F22, F26, F28, F34, F40, F48, F54, F63, F69, F71, F85, F90, F98, F106, F115, F101, F125, F132, F134, F140, F162, F163, F171, F173, F177, F182, M08, M13, M16, M25, M37, M49, M57, M62, M66, M72, M81, M89, M99, M105, M109, M114, M119, M123, M127, M135, M144, M147, M153, M160, M170, M175. Practice trial Identities: FACES identities F20 and M41) and calibrated to fit each participant’s individual screen in Testable (https://www.testable.org/ (accessed on 8 September 2024)), where the experiment was programmed and hosted. The emotions on the faces are recognizable with high accuracy [29,31]. Faces were fitted with opaque and clear masks using Adobe Photoshop CS6. To mimic real mask positioning, the masks were fitted on each face to horizontally span the edges of each cheek and vertically span the bridge of the nose to the bottom of the chin. The clear mask design followed an approved FDA model. Sample stimuli are displayed in Figure 1a.

### 2.3. Design and Procedure

This study was a repeated measures design with three factors: Face Orientation (2: Upright, Inverted), Mask (3: Opaque, Clear, No Mask), and Emotion (2: Happy, Neutral). Face Orientation manipulated whether the faces were displayed in upright or inverted orientation. Mask manipulated whether the lower face was fully visible (No Mask condition), largely visible (Clear Mask condition) or fully occluded (Opaque Mask condition). Emotion manipulated whether the faces displayed Happy or Neutral facial expressions. Emotional sentences generated from 20 general sentence themes (previously validated [15,16,17,18,31]) were paired with the faces to provide congruent emotional context (for happy faces, the sentences stated that the protagonist had experienced a positive event (e.g., “She aced her important physics test”), while for neutral faces, the sentences stated that the protagonist had experienced a neutral event (e.g., “She marked the important physics test”). Sentence pronouns always matched the face gender). We included these contextual sentences to help bridge to our previous work first demonstrating impairments in empathic ratings for masked faces [15] and to increase ecological validity of the situation, given that emotional expressions are rarely encountered without an affective context.

Mask and Emotion were intermixed within blocks while Face Orientation was blocked.

An example trial is illustrated in Figure 1b. Trials started with a presentation of an emotionally congruent sentence in a duration of 4000 ms. Then, a fixation cross was shown for 200 ms, after which a face wearing no mask, an opaque mask, or a clear mask and bearing a happy or neutral facial expression was presented for 2000 ms. Two rating screens followed the presentation of the face. The first screen asked participants to use a Likert scale ranging from 1 (very little) to 9 (extreme) to rate how much empathy they felt for the individual. The second screen asked participants to use a Likert scale ranging from 1 (very negative) to 9 (very positive) to rate the valence of the emotion they were feeling. These ratings corresponded to the amount of shared emotion, or affective empathy, and the qualia of that shared emotion with the protagonist (e.g., negative or positive [15,17,19,32]).

The task consisted of 240 trials divided into 10 blocks presented in random order, with 5 blocks displaying upright faces and the remaining 5 blocks displaying inverted faces. Each block presented an equal number of male and female faces, and an even representation of all emotion and mask conditions. Trial order within the blocks was randomized. An assignment of face identity to mask condition was counterbalanced between participants such that no participant saw the same face identity wearing two different mask conditions (e.g., for a single participant, face identity 1 would always be shown wearing an opaque mask). Each face identity was shown an equal number of times as upright and as inverted. Six practice trials were run at the start. The experiment took about 1 h to complete.

## 3. Results

The original data presented in this study are openly available in the Open Science Framework for the 116 of our 125 participants who consented to online data sharing at https://osf.io/b24wm/ (accessed on 8 September 2024). Mean ratings of empathy and valence were calculated for each Face Orientation, Emotion, and Mask condition and for each participant. These ratings were entered into two repeated measures ANOVAs with factors of Face Orientation (2; Upright, Inverted), Emotion (2; Neutral, Happy), and Mask (3; No Mask, Clear Mask, Opaque Mask), run separately on mean empathy and valence ratings. All follow-up *t*-tests were paired, two-tailed, and Bonferroni corrected for multiple comparisons. Greenhouse–Geisser corrected statistics and degrees of freedom are reported when Mauchly’s test indicated that violations of Sphericity had occurred.

To remind, we expected to replicate previous findings (e.g., [15,17]) showing reduced empathy ratings for masked relative to unmasked faces. Further, if this reduction in empathy was due to the disruption of configural face processing by face occlusion via masks, we expected to find larger inversion effects for unmasked faces than for occluded faces wearing masks (both opaque and clear). This follows from our hypothesis that configural processing of unmasked faces should be the most affected by face inversion.

### 3.1. Empathy

**Figure 2:**
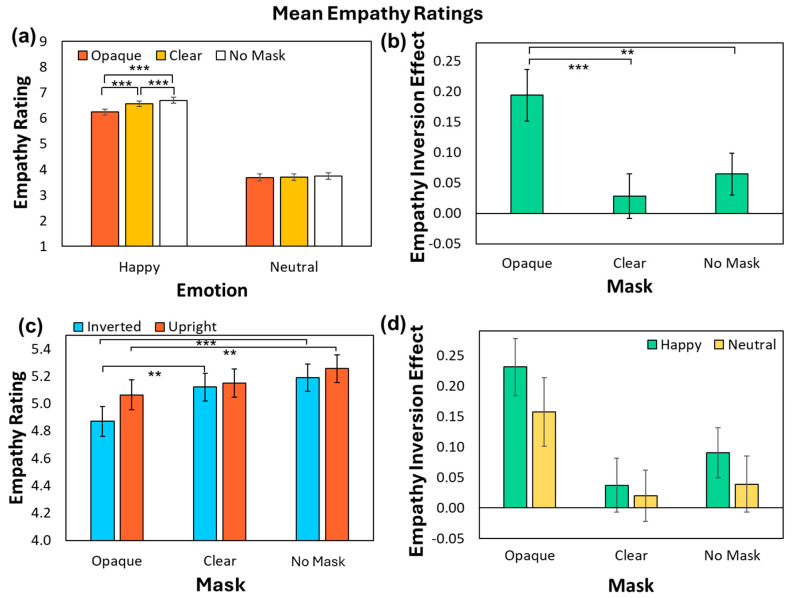
(**a**) Mean empathy ratings for each Emotion condition as a function of Mask. (**b**) Mean empathy rating inversion effect (upright empathy-inverted empathy) for each Mask condition. (**c**) Mean empathy ratings for Mask type as a function of Face Orientation. (**d**) Mean empathy rating inversion effect for each Mask and Emotion condition. Error bars represent Standard Error of the mean. Note: ** denotes *p* < 0.01, *** denotes *p* < 0.001.

Figure 2 illustrates mean empathy ratings as a function of Emotion and Mask conditions. As shown in Figure 2a, and replicating our previous work, significant main effects of Emotion (*F*(1, 124) = 492.94, *p* < 0.001, *MSE* = 5.92, *ηp*^2^ = 0.80) and Mask (*F*(1.42, 175.52) = 492.94, *p* < 0.001, *MSE* = 5.92, *ηp*^2^ = 0.80) indicated that participants reported more empathy in the Happy condition than the Neutral condition and for unoccluded faces compared to masked faces (Opaque vs. Clear and No Mask conditions). A significant two-way interaction between Mask and Emotion (*F*(1.55, 191.98) = 39.97, *p* < 0.001, *MSE* = 0.174, *ηp*^2^ = 0.24) was driven by a main effect of Mask in the Happy condition (*F*(1.36, 168.44) = 54.92, *p* < 0.001, *MSE* = 0.37, *ηp*^2^ = 0.31) but not in the Neutral condition (*F*(1.73, 213.93) = 1.97, *p* = 0.15, *MSE* = 0.12, *ηp*^2^ = 0.02). In the Happy condition, participants reported empathizing less when protagonists wore opaque masks compared to no masks (*t*(124) = −8.61, *p* < 0.001, *SE* = 0.053, *d =* −0.77) or clear masks (*t*(124) = −6.27, *p* < 0.001, *SE* = 0.050, *d =* −0.56), and when protagonists wore clear masks compared to no masks (*t*(124) = −5.64, *p* < 0.001, *SE* = 0.025, *d =* −0.51).

Figure 2b shows the effects of face inversion on empathy ratings as a function of mask. The ANOVA indicated a significant main effect of Face Orientation (*F*(1, 124) = 8.96, *p* = 0.003, *MSE* = 0.38, *ηp*^2^ = 0.067), with participants reporting overall more empathy when the faces were upright compared to when they were inverted. As illustrated in Figure 2b, there was also a significant interaction between Face Orientation and Mask (*F*(1.86, 230.69) = 12.28, *p* < 0.001, *MSE* = 0.083, *ηp*^2^ = 0.090). Paired *t*-tests comparing the magnitude of the inversion effect (i.e., a difference in ratings between upright and inverted conditions) for each mask condition indicated that face inversion lowered empathy ratings for opaque mask wearers more than for clear mask wearers (*t*(124) = 4.60, *p* < 0.001, *SE* = 0.036, *d* = 0.41) and no mask wearers (*t*(124) = 3.35, *p* < 0.01, *SE* = 0.039, *d* = 0.30), which did not differ (*t*(124) = −1.20, *p* = 0.70, *SE* = 0.030, *d =* −0.11 As illustrated in Figure 2c, the larger inversion effect for the Opaque compared to No mask condition was driven by reduced empathy ratings for faces wearing opaque masks in both the upright (*t*(124) = −4.79, *p* = 0.001, *SE* = 0.040, *d* = −0.43) and inverted conditions (*t*(124) = −8.91, *p* < 0.001, *SE* = 0.040, *d =* −0.72). In contrast, the larger inversion effect for the Opaque compared to Clear mask condition was driven by reduced empathy in the inverted condition only (*t*(124) = −6.54, *p* = 0.001, *SE* = 0.038, *d* = −0.59). No other effects were significant (Emotion x Face Orientation (*F*(1, 124) = 1.40, *p* = 0.24, *MSE* = 0.15, *ηp*^2^ = 0.011; Emotion x Mask x Face Orientation, (*F*(1.90, 235.45) = 0.44, *p* = 0.65, *MSE* = 0.062, *ηp*^2^ = 0.003). Thus, we found no evidence that the inversion effect was affected by emotion, as depicted in Figure 2d.

Thus, participants overall empathized more when faces were presented in the upright orientation and with happy compared to neutral faces. Empathy ratings were disrupted by face inversion, especially when those faces wore opaque masks.

### 3.2. Valence

**Figure 3:**
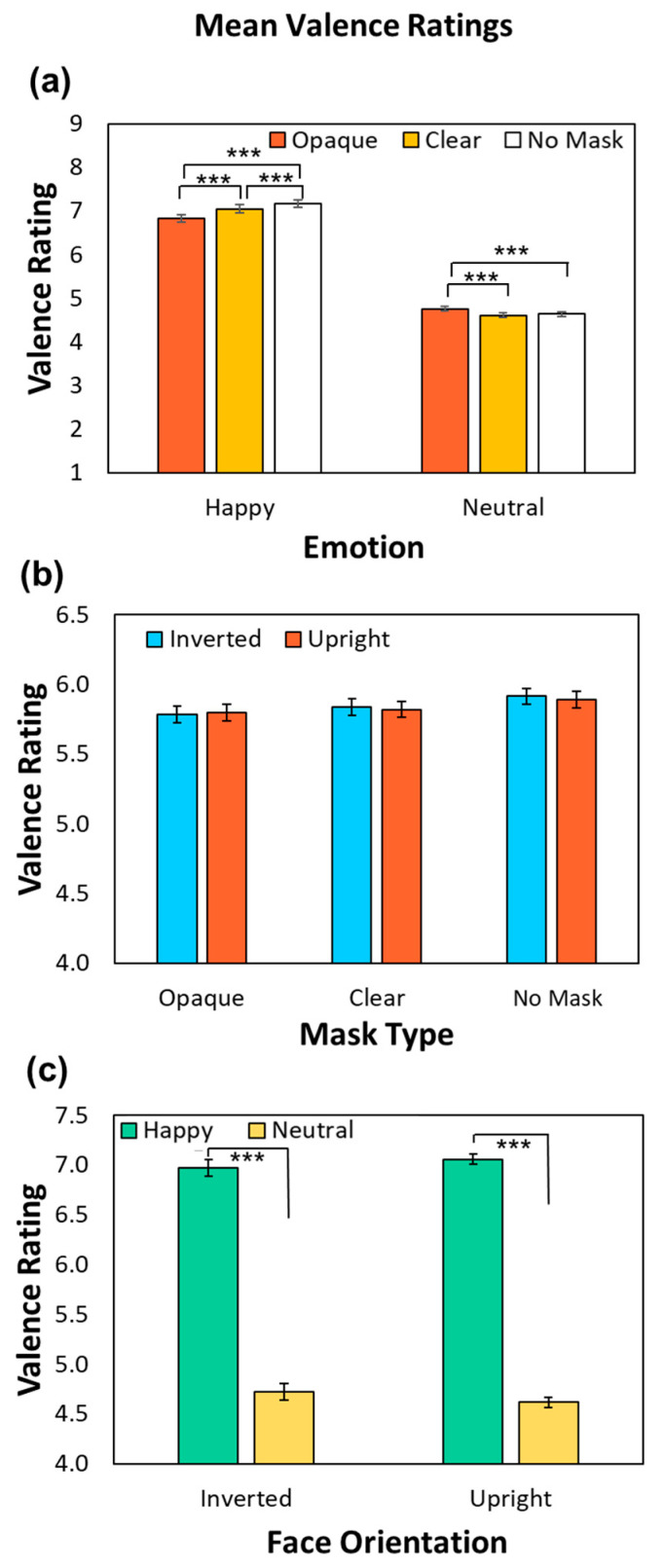
(**a**) Mean valence ratings for each Emotion as a function of Mask. (**b**) Mean valence ratings for each Mask Type as a function of Face Orientation. (**c**) Mean valence ratings for each Face Orientation as a function of Emotion. Error bars depict Standard Error. Note: *** denotes *p* < 0.001.

Figure 3 illustrates mean valence ratings as a function of Emotion (Figure 3a), Mask Type (Figure 3b), and Face Orientation (Figure 3c). The data were overall similar to empathy ratings. Significant main effects of Emotion (*F*(1, 124) = 835.57, *p* <.001, *MSE* = 2.47, *ηp*^2^ = 0.87) and Mask (*F*(1.63, 202.57) = 17.16, *p* < 0.001, *MSE* = 0.12, *ηp*^2^ = 0.12) indicated that participants reported more positive valence in response to Happy compared to Neutral protagonists and less positive valence ratings for protagonists wearing both opaque (*p* < 0.001) and clear (*p* < 0.001) masks compared to those wearing no masks. As depicted in Figure 3a, an interaction between Emotion and Mask (*F*(1.72, 213.20) = 78.65, *p* < 0.001, *MSE* = 0.11, *ηp*^2^ = 0.39) indicated a larger impact of face masks on Happy trials, though there was a main effect of Mask for both Happy (*F*(1.60, 198.00) = 49.96, *p* < 0.001, *MSE* = 0.12, *ηp*^2^ = 0.29) and Neutral trials (*F*(2, 248) = 39.33, *p* < 0.001, *MSE* = 0.038, *ηp*^2^ = 0.24). In Happy trials, participants reported experiencing less positive valence when protagonists wore opaque masks compared to no masks (*t*(124) = −8.76, *p* < 0.001, *SE* = 0.039, *d =* −0.78) or clear masks (*t*(124) = −5.78, *p* < 0.001, *SE* = 0.039, *d =* −0.52), and when protagonists wore clear masks compared to no masks (*t*(124) = −4.82, *p* < 0.001, *SE* = 0.025, *d =* −0.43). In Neutral trials, participants reported experiencing more positive emotion when protagonists wore opaque masks compared to no masks (*t*(124) = 6.70, *p* < 0.001, *SE* = 0.018, *d* = 0.60) and clear masks (*t*(124) = 7.79, *p* < 0.001, *SE* = 0.019, *d* = 0.70), which did not differ (*t*(124) = −1.76, *p* = 0.25, *SE* = 0.016, *d =* −0.16).

As seen in Figure 3b, a main effect of Face Orientation was not significant (*F*(1, 124) = 0.18, *p* = 0.67, *MSE* = 0.17, *ηp*^2^ = 0.001); however, this was qualified by a two-way interaction between Face Orientation and Emotion depicted in Figure 3c (*F*(1, 124) = 40.51, *p* < 0.001, *MSE* = 0.088, *ηp*^2^ = 0.25). While there were main effects of Emotion on valence ratings for both inverted (*F*(1, 124) = 748.42, *p* < 0.001, *MSE* = 1.27, *ηp*^2^ = 0.86) and upright faces (*F*(1, 124) = 866.58, *p* < 0.001, *MSE* = 1.29, *ηp*^2^ = 0.88), with participants reporting more positive valence for upright faces, the effect was numerically larger for upright faces. No other effects were significant (Face Orientation and Mask (*F*(2, 248) = 1.29, *p* = 0.28, *MSE* = 0.046, *ηp*^2^ = 0.010; Emotion, Mask, and Face Orientation (*F*(2, 248) = 2.11, *p* = 0.12, *MSE* = 0.043, *ηp*^2^ = 0.017).

If reduced empathy for masked faces was driven by disrupted configural face processing, we expected that face inversion would lower empathy ratings for unoccluded faces more than for masked faces, as unoccluded faces benefit from configural processing in the upright orientation. In contrast, while face inversion reduced empathy ratings, it did so most for the protagonists wearing opaque masks compared to those wearing clear masks or no masks.

## 4. Discussion

The present study investigated the underlying mechanisms involved in impaired positive empathy for occluded faces wearing masks. Given that wearing face masks potentially disrupts configural face perception [11,20], here, we investigated whether mask-related empathy impairments could also be attributed to disrupted face configural processing. Participants rated their empathy for faces wearing opaque, clear, or no masks. Half of the faces were viewed in an upright orientation while the other half were viewed in an inverted orientation, which is a common manipulation theorized to disrupt configural face perception [22,23,24,25]. Our results indicated that face inversion and face occlusion by masks reduced empathy and lowered valence ratings. We found a similar effect in empathy reduction for clear and opaque masks, adding to a body of work illustrating that facial occlusion leads to social impairments in a variety of domains (e.g., [7,10,11,12,14]) and suggesting that wearing clear masks, which preserve visual access to the face, does not fully alleviate social barriers created by face masks (but see [7,15] for the positive effects of clear masks). Critically, however, face inversion reduced empathy ratings the most for faces wearing opaque masks compared to faces wearing no masks. We next discuss key points relating to these findings.

First, our results indicated that overall face inversion resulted in reduced ratings of empathy and minimized the impact of emotion condition on valence ratings. One explanation for this result is that empathic responding is reduced by face inversion because face inversion disrupts configural face perception, which is needed for the extraction of the face’s social value [22,23,24,25]. This dovetails with data showing that both configural processing [33] and empathy [34,35] are stronger for own-race faces, and with reports of altered affective empathy in autism spectrum disorder [36], which has been associated with deficits in configural face processing [37]. Alternatively, face inversion could also disrupt empathy for other reasons, such as acting to depersonalize the individual. Thus, future work is needed to understand if a similar impairment in empathy is observed when other common configural processing manipulations such as the Composite Face Effect [38] or Part–Whole Task [39] are used.

Second, and more critically, we found that face inversion impacted empathy ratings more for faces wearing opaque masks than those wearing clear or no masks, which remained less impacted by inversion. In contrast, the classic conceptualization of face inversion [23] predicts that inversion should lower empathy ratings for unmasked faces the most, given their original intact configural perception when upright. There is some support for this notion, with larger inversion effects for recognition of unmasked compared to masked faces [12,20]. However, a recent study by Fitousi et al. [11] found that inversion impacted gender discrimination more for masked than for unmasked faces, while producing no impact on emotion or age discrimination. Our work dovetails with this result to suggest that while face masks appear to impair configural face processing, configural processing deficits only explain part of the mask-related social impairments.

Indeed, within the classic conceptualizations of configural processing (e.g., [21]), it is possible that processes such as face recognition may rely strongly on configural processes, while the extraction of other social and emotional face information may depend more on part-based information, given that certain face parts have been associated with relaying visually diagnostic information for various emotions (e.g., eyes for sadness, and mouth for disgust; [40,41,42,43]). This is in line with alternative models of face processing in which face inversion is seen as impacting face perception in a quantitative instead of qualitative manner [44]. For example, Sekuler et al. [44] manipulated linear classification images of upright and inverted faces and found that participants used the eye-region of both upright and inverted faces, with no evidence of a qualitative change in processing style with inversion.

It is also possible that the classic idea of configural face processing is most highly related to low-level face properties such as face detection or identification, while the emergent properties of a face, such as those that signal its social, emotional, or individual value, may further require information from part-based processing alongside integration of perceptual, social, and contextual aspects. For example, studies in which faces are stripped from their typical social context, including low-level visual differences within a face, hairline, emotion, and task relevance show that under these conditions, faces cease to attract attention preferentially (e.g., [45,46]). Thus, configural face processing may only contribute partially to the computation of emergent face properties like emotion understanding and empathic responding. The results of the present work suggest that while visual access to faces facilitates empathic responding, configural face processing may not contribute to this process only when faces are masked.

If disrupted configural processing cannot fully explain the masked empathy impairment, it is possible that the processing deficit stems from difficulty in recognizing masked emotional expressions due to reduced visual access to the face. Indeed, we have previously demonstrated for upright faces that reducing visual access by occluding a face with an opaque mask leads to emotion recognition deficiencies, while clear masks restore both visual access and emotion recognition ([15] and replicated here). However, empathy ratings are reduced for faces wearing both opaque and clear face masks, which is problematic for the interpretation that the empathy deficit is driven by reduced visual access to the face. However, altered emotion recognition could account for the pattern of data we observed here for inverted faces, as inversion uniquely lowered empathy ratings for faces with opaque masks, which in turn are the most affected by reduced visual access, and thus are susceptible to lowest emotion recognition [14,17]. In other words, while impaired emotion recognition does not seem to fully explain the impaired empathy for upright masked faces, it may explain why the inverted faces with opaque masks resulted in very low empathy ratings. This would suggest different processing invoked for upright and inverted masked faces.

A few models of face perception, such as Rakover’s [47] face–scheme incompatibility (FSI) model, provide a rationale for upright and inverted processing differences. The FSI model proposes that upright and inverted faces both gain meaning through comparison to upright and inverted “schemes” (constructs portraying general face structure), but that upright schemes are more efficient, given more familiarity with upright faces in interactions. According to Rakover [47], inverted eyes are typically compared to upright schemes since they look relatively similar when inverted, and it is only through the context of other face parts that appear more clearly inverted (e.g., the nose) that schemes are reconciled and switched (e.g., from upright to inverted). Contextualizing our findings within this model, inversion of opaque masked faces would invoke an upright face scheme to process inverted eyes without the other face features visible to signal that inversion has occurred—a problem that would be solved for the unmasked or clear masked faces, which have other visible face features the system can use to reconcile the conflict. This could worsen the extraction of emotional information, resulting in lower empathy specifically for inverted faces wearing opaque masks. In the future, this could be tested by comparing inverted faces wearing different types of face occlusions (e.g., mouth vs. eye occlusion), leaving other face features like the nose and mouth unoccluded, which are more clearly identified as inverted than eyes [47].

Another possible future direction would be to study the impact of face masks on empathy for emotions other than happiness. We have previously found that empathy was intact for sad faces covered with face masks [12], but it is possible that empathy for other emotions like disgust or anger may be impaired as well. Given that clear masked faces produce intact emotional inferences but still impair empathy ratings [12], it seems unlikely that lowered emotional recognition is the only driving force behind lowered empathic ratings for masked faces. Inclusion of more emotional expressions could help test this idea. That is, there are large differences in how much face masks impact recognition of different basic emotions (e.g., 40% recognition reduction for disgust vs. 10% recognition accuracy for fear), which could offer predictions about how much empathy for that emotion should be impaired if emotion recognition is the driving mechanism (e.g., [13,14]).

In summary, in this study we found that visual face occlusion by masks and face inversion lead to reduced empathy ratings, with face inversion particularly exacerbating the negative impact of opaque masks on empathy ratings. These findings suggest that impaired empathy for masked faces is not fully explained by impaired facial configural processing as conceptualized by classic models, and suggest that facial occlusion may affect social extraction of face values only partly via disruption of configural face processing.

## Data Availability

The original data presented in this study are openly available in the Open Science Framework for the 116 of our 125 participants who consented to online data sharing at https://osf.io/b24wm/ (accessed on 8 September 2024).
